# Sensorineural Hearing Loss in Dengue: A Pilot Study

**DOI:** 10.22038/ijorl.2020.39874.2314

**Published:** 2021-05

**Authors:** Kapil Soni, Gopal-Krishana Bohra, Nithin-Prakasan Nair, Darwin Kaushal, Sourabha-Kumar Patro, Amit Goyal

**Affiliations:** 1 *Department of Otorhinolaryngology, All India Institute of Medical Sciences, Jodhpur.*; 2 *Department of Medicine, All India Institute of Medical Sciences, Jodhpur. *

**Keywords:** Dengue fever, Hearing loss, Hearing test

## Abstract

**Introduction:**

Association of hearing loss has been found with a couple of febrile illnesses. Dengue fever is an arboviral febrile illness that is transmitted by Aedes mosquito. A case of sensorineural hearing was documented in the literature recently in dengue haemorrhagic fever. We are aiming to find if hearing loss occurs in dengue patients.

**Methods and Methodology:**

We assessed the hearing of ten patients diagnosed with Dengue fever from August 2018 to October 2018, prospectively. Patients who had a prior history of hearing loss or chronic suppurative otitis media were excluded from the study. Brief history, clinical examination and audiological assessment were made for all patients. All patients were followed up for three months with repeat audiological evaluation.

**Results:**

Two patients complained of hearing loss after the onset of fever. They had a bilateral mild sensorineural hearing loss on audiological evaluation. One other patient was found to have bilateral high-frequency hearing loss although there was no complaint of hearing impairment. On three months follow up, both patients had bilateral mild sensorineural hearing loss with no improvement.

**Conclusion:**

Hearing loss in dengue fever, even though being mild in nature is irreversible. The cause of hearing loss in dengue is yet to be found. For the definitive association of hearing loss in dengue fever further studies are required.

## Introduction

Dengue is an important arboviral infection affecting humans which results in high morbidity and mortality. It is estimated that 50 million infections per year occur worldwide, across approximately 100 countries (1,2). Dengue fever is an endemic illness in India. Dengue fever had a predominant urban distribution a few decades earlier. But it is now also reported from peri-urban as well as rural areas (3,4). Dengue virus belongs to the family Flaviviridae, is transmitted by the Aedes aegypti mosquito (1,2,5). Following an incubation period of 3-7 days, symptoms start. Dengue fever follows three phases: an initial febrile phase, a critical phase and a spontaneous recovery phase. Patients generally recover after an initial febrile phase without much complications (1,2,6).

In Dengue Haemorrhagic fever, the initial symptoms are the same but generally present with worsening of signs on the third or fourth day. Patient presents with agitation, lethargy, tachycardia, hypotension, spontaneous bleeding manifestations, cyanosis and hypothermia (1,2,6).

Detection of non-structural protein 1(NS1 Antigen) by enzyme-linked immunosorbent assay (ELISA) is sufficient for confirmatory diagnosis (7). The general ENT manifestations in Dengue fever are rhinitis, sore throat, nasal obstruction, otalgia, dizziness, bleeding manifestations like nasal bleeding, gingival bleed, and salivary glands alterations (6). Patients who have hearing loss following viral infection is often sensorineural, sometimes mixed (measles infection, CMV) or conductive (measles infection). The proposed hypothesis for sensorineural hearing loss includes direct viral damage, and the patient’s immune system directed damage to the peripheral auditory system (like in Cytomegalovirus (CMV)) (8).

A study done in Boston revealed that hearing loss was detected in 63% of patients who were diagnosed to have antibodies against rubella, mumps, varicella-zoster virus, CMV, or influenza. In another Greek study of 262 patients with unilateral sudden hearing loss, only in 27% of patients, there were antibodies against Epstein–Barr virus (EBV), herpes simplex (HSV) type I and II virus, or CMV(4).

There is no definite association of hearing loss proved with dengue in the medical literature. There is only one case report of sensorineural hearing loss associated with dengue haemorrhagic fever in which hearing loss is reported. Association of hearing loss with dengue is an enigma. This paper aims to explore the association of dengue with hearing loss.

## Methods and Methodology

A prospective study of 10 patients diagnosed with dengue fever was done. Institute ethics committee clearance was obtained prior to start of this study (No. AIIMS/IEC/2018/1423). The patient presented in Department of Medicine with NS1 antigen-positive dengue fever were included in the study from September 2018 to October 2018. After October 2018, due to change in weather we did not get any NS1 antigen positive dengue fever patient. All patients from age 16 to 65 years were included in the study. Patients who had a prior history of chronic otitis media or hearing loss were excluded from the study. We excluded the patients with hypothyroidism, diabetes mellitus and neurological diseases. We also excluded patients with a history of exposure to noise and ototoxic drugs. A brief history, along with clinical examination, including otoscopy and tuning fork test were done as soon as the patients were diagnosed with dengue. A complete audiological evaluation was done, including pure tone audiometry with average of 500Hz, 1000Hz, 2000Hz, Tympanometry, Brainstem evoked response audiometry, and Steady-state auditory response. Baseline evaluation of fever was also done simultaneously, including complete hemogram, serology workup for other febrile illnesses including malaria, chicken guinea, typhoid, scrub typhus etc. Patients who had hearing loss were treated as per standard protocol. Patients were followed up to 3 months with repeat audiological evaluation. The patients were re-evaluated at three months, to confirm hearing loss and to assess the reversibility.

## Results

Ten patients were included in the study and were assessed prospectively. Following confirmation of dengue with NS1 antigen positivity, patients were analyzed clinically and with the audiological test. The Majority of patients were male (8 out of 10). The mean age of patients was 29 years. The Mean platelet count of patients (lowest) was 50960 per cubic millimetre. The Mean Haemoglobin of patients was 13.6 g/dL. The Mean haematocrit value was 42.35 %. 

The following table shows the characteristics of patients who were included in the study. (Table.1)

**Table 1:** Patient characteristics who were included in study

**Patient**	**Sex**	**AGE**	**Complaints Of Hearing Loss**	**Tinnitus** **History**	**Pta Rigth** **Ac/Bc**	**Pta Left Ac/Bc**	**Tympanomtery**	**Dpoae**	**ABR**	**Right Assr** **0.5-1-2-4** **Khz (Db)**	**Left Assr** **0.5-1-2-4** **Khz (Db)**	**Teoae**	**Hearing Status**
1	Male	18	NO	NO	15/10	15/10	A	PASS	Wave forms noted at 30 dB bilaterally	15-10-12-13	10-15-13-15	Pass	B/L Normal Hearing
2	Female	50	YES	NO	32/30	33/30	A	REFER	Wave forms noted at 60 dB bilaterally	35-30-25-38	40-30-35-30	Refer	B/L Senosorineural Hearing Loss
3	Female	30	YES	NO	30/26	30/26	A	REFER	Wave forms noted at 60 dB bilaterally	38-40-35-40	40-35-35-40	Refer	B/L Senosorineural Hearing Loss
4	Female	21	NO	NO	17/12	18/12	A	PASS	Wave forms noted at 30 dB bilaterally	10-18-20-18	10-15-15-20	Pass	B/L Normal Hearing
5	Male	65	NO	YES	20/15	20/12	A	PASS	Wave forms noted at 60 dB bilaterally	20-20-35-40	10-20-20-40	Pass	B/L High Frequency Hearing Loss
6	Male	26	NO	NO	15/10	15/10	A	PASS	Wave forms noted at 30 dB bilaterally	10-15-15-10	12-10-18-18	Pass	B/L Normal Hearing
7	Male	23	NO	NO	17/15	13/10	A	PASS	Wave forms noted at 30 dB bilaterally	15-20-20-18	15-15-18-15	Pass	B/L Normal Hearing
8	Male	21	NO	NO	15/10	17/12	A	PASS	Wave forms noted at 30 dB bilaterally	12-15-12-15	15-12-12-18	Pass	B/L Normal Hearing
9	Male	16	NO	NO	18/12	18/14	A	PASS	Wave forms noted at 30 dB bilaterally	15-20-14-18	15-15-14-20	Pass	B/L Normal Hearing
10	Male	20	NO	NO	18/10	17/10	A	PASS	Wave forms noted at 30 dB bilaterally	20-15-15-20	20-15-20-20	Pass	B/L Normal Hearing

Two patient’s complained about hearing loss 2 to 3 days following the onset of fever. On audiological evaluation, both patients had bilateral mild sensorineural hearing loss. Another patient aged 65 years had bilateral high-frequency hearing loss with complaints of tinnitus too. All these findings are overlapping with presbycusis. We may not consider this hearing loss is caused by dengue fever. 

All other patients had normal hearing sensitivity. All patients had normal tympanometry. Two patients with hearing loss had otoacoustic emissions referred. All patients had normal BERA waveforms. ASSR also corresponded to the findings. Patients who had hearing loss are being summarised below

***Case 1***

The patient was a 50-year-old female who was brought to medicine emergency department with complaints of fever with chills and rigors. The patient was admitted and evaluated and was found to be NS1 antigen positive. She did not give any history suggestive of bleeding manifestations. On serial hemograms, the patient was found to have low platelet counts falling as low as 70000. She complained of bilateral impairment of hearing, two days after the onset of fever. On audiological evaluation, she had mild sensorineural hearing loss both ears. She was treated on high dose steroids in line with sudden sensorineural hearing loss. For dengue fever, she was managed conservatively. On three months follow up, repeat pure tone audiogram was done, which showed no improvement.

***Case 2***

The patient was a 30-year-old female who was brought to medicine emergency with complaints of fever for seven days which was associated with chills, rigors, myalgia and headache. She was found to be NS1 antigen positive. She did not complain about any bleeding manifestation but had low platelet counts, as low as 25000. She complained of hearing loss, three days after the onset of fever. On further evaluation, she also had bilateral mild sensorineural hearing loss. She was also treated as per standard protocol for sudden sensorineural hearing loss. On three months follow up, repeat audiogram was done which showed no improvement.

***Case 3***

The patient was a 65-year-old male who was brought to medicine emergency with complaints of fever for five days associated with chills, rigors, myalgia and headache. On further evaluation, the patient was found to be NS1 antigen positive. He did not have any prior history of hearing loss before the episode of fever. The patient had low platelet counts, lowest being 65000. On Pure Tone Audiogram, patient was found to have bilateral high-frequency hearing loss. On follow up after three months, repeat pure tone audiogram showed no improvements.

Patients with hearing loss had fewer platelet counts as compared to patients without hearing loss. We couldn’t find any relation with temperature/haemoglobin/platelet counts/haematocrit.

Patients with mild sensorineural hearing loss were started on neurotropic drugs. On three months of follow up, repeat audiological evaluation showed a similar picture.

## Discussion

We could find only one case report, reporting a case of sensorineural hearing loss associated with dengue haemorrhagic fever in literature (9). Two patients in our study developed mild sensorineural hearing loss which was irreversible at three months follow up. One patient developed high frequency irreversible sensorineural hearing loss.

The hearing loss following the onset of dengue fever cannot be ignored. We searched all causes of hearing loss, but only correlated factor was the dengue fever. As our sample size is small, a definite association of hearing loss with dengue fever cannot be confirmed. This study may enlighten further research to clarify the association between hearing loss and dengue fever. 

As the age range of patients in our study is wide, the possibility of asymptomatic presbycusis cannot be ruled out in two patients. 

Still, we had one young patient in our study who had hearing loss. Moreover, all three patients complaint about new-onset hearing loss following the onset of fever.

We repeated the hearing assessment after three months with a pure tone audiogram in all cases to identify any delayed onset nature of hearing in all patients and if hearing loss is reversible in patients who had hearing loss.

There are few viral infections which can lead to hearing loss, for example, mumps and cytomegalovirus. Hearing loss has also been sporadically reported in diseases with flaviviruses, including West Nile virus, Zika virus (9-12). 

Scrub typhus is also said to be associated with hearing loss (13). The hearing loss in scrub typhus was found to be reversible.

The symptoms of dengue haemorrhagic fever are generally non-specific. Patients typically present with high-grade fever with features suggestive of upper respiratory infection. Patients may also present with frontal headache, retro-orbital pain or bleeding manifestation, which may be associated with low platelet count. It seems that symptoms like ear pain (36.6%), vertigo (20%) and tinnitus (6%) may appear in dengue patients (6,14). One of our patient also had complained of tinnitus. However, hearing loss has not been reported as a manifestation of dengue in literature.

The mechanism of hearing loss by different viruses vary. The most popular hypothesis is by direct damage or immune-mediated damage to inner ear structures (8). The virus invades the fluid spaces and soft tissues of the cochlea (cochleitis) or invades cochlear nerve (neuritis), leading to damage of the inner ear apparatus. Sometimes, there will be reactivation of a latent virus within tissues of the inner ear, leading to its damage (8,15).

Patients with hearing loss had low platelet counts too. But still, we cannot establish any correlation based on just two cases. We could not find any relationship with other haematological parameters.

We used a battery of investigations to identify the type of hearing loss and to compare and confirm our findings. We considered doing ASSR as it will be beneficial for assessing patients with profound hearing loss (16).

We need to analyze a large sample of dengue patients with a longer follow up to establish the association of hearing loss and dengue and the reason for hearing loss in dengue patients.

## Conclusion

A couple of febrile illnesses are associated with hearing loss. Dengue is an arboviral febrile illness which may also lead to hearing loss. As we have two patients with dengue who had objective and subjective hearing loss. Still, further studies and long term follow up is required to prove the association of dengue fever with sensorineural hearing. 

The reason for hearing loss in dengue fever is also unknown and need to be investigated. The patients in our study had no improvement on follow up with pure tone audiogram. 

It may indicate that the hearing loss in dengue patients may be irreversible. Large sample size has to be analyzed and had to be followed up for a more extended period to establish the same.
